# Practical considerations for using concentrated U200 insulin in automated insulin delivery systems

**DOI:** 10.3389/fendo.2025.1692589

**Published:** 2025-10-28

**Authors:** Brynn E. Marks, Patricia Y. Chu, Neha Parimi, Risa M. Wolf, Mai Tran, Cari Berget

**Affiliations:** ^1^ Department of Pediatrics, Division of Endocrinology and Diabetes, Children’s Hospital of Philadelphia, Philadelphia, PA, United States; ^2^ Perelman School of Medicine at the University of Pennsylvania, Philadelphia, PA, United States; ^3^ Division of Endocrinology, Diabetes and Metabolism, Department of Medicine, University of Pennsylvania Perelman School of Medicine, Philadelphia, PA, United States; ^4^ Department of Pediatrics, Division of Endocrinology, Johns Hopkins School of Medicine, Baltimore, MD, United States; ^5^ Division of Endocrinology, Children’s National Hospital, Washington, DC, United States; ^6^ Barbara Davis Center for Diabetes, University of Colorado, School of Medicine, Aurora, CO, United States

**Keywords:** diabetes mellitus, type 1, insulin infusion systems, automated insulin delivery, concentrated insulin, patient safety, clinical protocols

## Abstract

The use of automated insulin delivery systems (AID) is standard of care for people with type 1 diabetes. However, the limited capacity of insulin pump cartridges, which can hold 1.6-3.0mL or the equivalent of 160–300 units of U100 insulin, can be a barrier to AID use for individuals with high total daily insulin (TDI) requirements. With the rising prevalence of obesity, expansion of AID use to type 2 diabetes, and trends towards smaller cartridge volumes to decrease the size of devices, practical solutions to reduce barriers to AID use for those with high TDI requirements are needed. U200 concentrated rapid-acting insulin (U200) has a similar pharmacokinetic and pharmacodynamic profile to U100 insulin, provides the same dose of U100 insulin in half of the volume, and has been used off-label to facilitate AID use for those with high TDI needs. In this perspective piece we provide practical considerations for clinical implementation of U200 use in AID systems, including identification of candidates, unique considerations in filling pumps with U200 insulin, guidance on programming appropriate AID settings for the different algorithms, concepts to address in patient education, and recommendations for standardized documentation in the electronic health record.

## Introduction

Automated insulin delivery systems (AID) are the preferred treatment modality for type 1 diabetes (T1D) because use improves time in range (TIR), reduces hypoglycemia, and helps to ease the burden of diabetes (1, 2). In the last year, the results of clinical trials demonstrating the efficacy of AID in individuals with type 2 diabetes (T2D) have further expanded indications for AID use (1, 3, 4). However, AID remains underutilized (5). For persons with diabetes (PWD) with high total daily insulin requirements (TDI), one potential barrier is the limited capacity of insulin cartridges, which can hold between 1.6-3.0 mL or the equivalent of 160–300 units of U100 insulin (U100). Furthermore, there is variable insulin absorption with large boluses and many individuals experience leaking (6, 7). Given the increasing rates of obesity and insulin resistance in people with T1D (8, 9) and the expansion of AID to T2D, innovative solutions to improve access in individuals with high TDI requirements are needed.

Use of U200 concentrated rapid-acting insulin (U200) in AID (U200-AID) could facilitate uptake of AID in this population. U200 has a comparable pharmacokinetic and pharmacodynamic profile to U100 and provides the same insulin dose in half the volume, effectively doubling the capacity of insulin pump cartridges (10). While clinical use of U200-AID has increased, there are no prospective studies evaluating its safety or effectiveness. In our retrospective cohort study of 50 adolescents and young adults using Insulet Omnipod 5 or Tandem Control-IQ, we assessed changes in glycemia and use of insulin pump supplies after transitioning from U100 insulin to U200-AID (11). For this cohort with a baseline TDI of 102.6 units (U100), days between pump cartridge changes increased significantly from 2.2 to 3.0 days. Although the TIR goal of ≥70% was not reached, TIR improved from 44.6% to 48.9% despite no significant change in the number of user-initiated boluses per day. Improvements were greatest for those with baseline hemoglobin A1c (HbA1c) >8%. Importantly, time below range (<70mg/dL) did not meaningfully increase and no episodes of DKA or severe hypoglycemia were identified.

## Regulatory stance on the use of concentrated insulin in pumps

Insulin is classified as a high-alert medication because its complex dosing regimen and narrow therapeutic index place PWD at risk of significant harm if used erroneously (12). If U100 and U200 were to be confused in AID, this could lead to two-fold differences in the insulin dose delivered with the possibility of causing hypoglycemia, hyperglycemia, ketosis, or even diabetic ketoacidosis (DKA). The Food and Drug Administration (FDA) classifies insulin pumps and AID as Class II medical devices, a distinction that recognizes the need for special performance standards, post-market surveillance, and labeling requirements to ensure reasonable safety and effectiveness.

U200-AID is not currently FDA approved; in fact, the U200 package insert states it should not be used in insulin pumps. Although no statement regarding the use of U200-AID has been issued by the FDA, inferences as to what might be required for FDA approval can be made from existing FDA guidelines. To identify and mitigate potential hazards, human factors engineering should be used to understand how the system interface, usage environment, AID algorithm, and interactions between PWD and their caregivers may contribute to risk with U200-AID (13). FDA approval for U200-AID would require a submission with data demonstrating that the modified device is as safe and effective as the previous version. The availability of dedicated devices for U200-AID (cartridges, syringes, pumps) to reduce the risk of insulin delivery as much as possible may be needed (14). Regardless, U200-AID is often used off-label for those with high TDI requirements, and it is important to highlight practical considerations for the safe use.

## Candidate selection and cost considerations for U200-AID

### Candidates for U200-AID

Most insulin pump reservoirs are intended to provide sufficient insulin for 72-hours and hold 1.6-3.0mL, or 160–300 units of U100. Recognizing that the fill capacity is often less than what is listed and the inconveniences of needing to fill cartridges more frequently than every 72 hours, we consider using U200 for those using: 1.6mL cartridge with TDI >50 units/day, 2.0mL cartridge with TDI >65 units/day, or 3.0mL cartridge with TDI >95 units/day. Some insulin pump reservoirs, including the 3.0mL Medtronic 780G Extended Reservoir, are approved for 7-days of use. Individuals using this 7-day reservoir who require a TDI >40 units/day may benefit from U200. U200-AID should be avoided in those with smaller TDI given the known limitations of insulin delivery accuracy for smaller doses of insulin which have been observed across many different insulin pumps (15, 16).

### Insurance and cost considerations

Insurance and cost may play a role in access to U200. The cost of insulin and diabetes supplies varies according to healthcare system, country, pharmacy, insurance coverage, product tier, and manufacturer rebates. Most insured individuals do not pay the listed prices, and out-of-pocket costs vary according to which products are preferred for each insurance company. Another important cost consideration is the administrative time required to submit and follow-up on the prior authorizations (PA) and appeals needed to exceed the quantities of cartridges and insulin typically allowed by insurance.

While insurance coverage for U200 differs according to the insurance company and individual coverage plans, fortunately it is covered by many public and commercial insurance companies with a PA. Commonly noted requirements for PA approvals are high HbA1c levels and a high TDI relative to the insulin pump cartridge size. Providing a clear explanation of the high TDI and implications for AID use often supports PA approvals. For PWD who are uninsured or underinsured and those with low-income, many manufacturers have specific programs to reduce copays for insulin, which often include U200. For example, the Lilly Cares Program can reduce U200 copays to as little as $35/month (17, 18).

In July 2023, the average wholesale prices per 1000 units of insulin were: Humalog U100 vials: $80, Humalog U100 KwikPens: $127, and Humalog U200 KwikPens: $424 (19). The costs of U200 should be considered alongside the savings resulting from use of fewer insulin pump supplies after a recently published study noted that transitioning from U100-AID to U200-AID increased the number of days of insulin pump cartridge use (11). Unfortunately, average wholesale prices of insulin pump supplies are not readily available; however, the decrease in pump supplies required with U200-AID use should be factored into cost considerations. A PWD with a TDI of 150 units/day who uses Omnipod 5 (2.0mL cartridge) would require 3 fewer boxes of pods per month if using U200. If this same individual were using U200 in a Tandem X2 pump (3.0mL cartridge), they would require one fewer box per month.

The unique financial considerations for U200-AID users within public healthcare systems should also be considered. Public systems provide greater cost transparency and avert the additional costs incurred from contracts between insurers and pharmacy benefit managers. U200-AID may offer even greater cost savings in public health systems, which could help to further expand access to AID.

## Educating people with diabetes and clinicians to support safe use

Education for both clinicians and PWD is vital to ensure safe use of U200-AID. Clinicians must know how to adjust AID settings when switching from U100 to U200 and understand algorithm function sufficiently to recognize when a factory reset is necessary. PWD should understand the difference between U100 and U200, that different pump settings are needed for each insulin formulation, and the importance of informing medical and emergency personnel of U200 use. PWD should be encouraged to use the bolus calculator rather than manually bolusing to avoid accidental insulin over-doses.

Additionally, in cases of pump failure or emergencies where insulin needs to be administered as an injection, it is essential that PWD are informed that U200 must be given via insulin pen. U200 pens are calibrated to deliver half the volume of U100 pens, thereby delivering the same number of units. U200 users should be reminded that if they are using U200 insulin pens to deliver an injection, they would dial the same number of units on the pen whether they are using a U100 or U200 pen. Also, if PWD need to deliver insulin via syringe, they must avoid U200 and use U100 instead. However, the insulin doses derived from the pump settings need to be doubled with administration of U100. Therefore, PWD and their caregivers including school nurses should be provided with clear dosing instructions to avoid undertreatment and hyperglycemia.

## Documentation in the medical record

To ensure clear documentation of U200-AID use in the written or electronic medical record, practices should establish protocols that make this information readily visible in the PWD’s medical record. Suggestions for effective documentation include noting use of U200 in the list of medical problems, on the “Patient Care Coordination Note” and/or in the “specialty comments” section of the health record. All encounters with the endocrinology team should explicitly mention the use of U200-AID. This clarity is important for continuity of care, particularly for the school setting, during hospital admissions or in case of emergency situations or transfers of care.

Diabetes Medical Management plans for school and written documentation given to U200-AID users should provide guidance on how to transition between U100 and U200 delivered by AID and injections in case of an unexpected need to convert from U200 to U100. Schools and PWD should be instructed to contact the diabetes team to confirm doses in case the insulin delivery modality is changed.

## Filling insulin pump cartridges with U200 insulin

U200 rapid-acting insulin is currently only available in an insulin pen (Humalog KwikPen); it does not come in a vial. *Once an insulin pen has been used to fill a pump cartridge, the pen can no longer be used to inject insulin directly into the body because air bubbles will be present in the pen.*

When using an insulin pen to fill a pump cartridge, there are several factors to consider. The syringes provided with the insulin pump cartridges (rather than insulin pen needles) should be used to extract insulin from the pens and to fill the pump cartridge. When using an insulin vial to fill a pump cartridge, users are instructed to add air before drawing out insulin. No air should be added to the insulin pens because they are nearly completely full of insulin and too small to accommodate this additional pressure. Once the pump cartridge syringe has been inserted into the pen, insulin can be drawn into the syringe by pulling on the syringe plunger.

## Programming AID settings for use with U200

In general, the volume of insulin delivered should be cut in half when using U200 compared to U100 insulin in AID. When programming U200 pump settings for basal rates, carbohydrate ratios and correction/sensitivity factors, reduce all settings by at least 50%. Further dose reductions may be necessary for those with very high TDI due to decreased insulin leakage and/or improved insulin absorption when using U200. For example, if the basal rate is 2.0 units/hr with U100, it should be reduced to 1.0 unit/hr for U200. Likewise, a carbohydrate ratio of 1 unit: 5g and correction factor of 1 unit: 10 mg/dL on U100 should be reduced to 1 unit:10g and 1 unit: 20 mg/dL on U200, respectively.

When using AID, it is also important to consider how each algorithm functions to understand how to safely use U200 with the automated features of each system. Below are key considerations for U200-AID with each commercially available system in the U.S. Please see **Table 1** for additional recommendations about adjustments to AID system-specific safety settings, including maximum basal rate and bolus settings. For the AID systems described in this manuscript, the programmed maximum basal rate and bolus do not influence algorithm determined insulin delivery. Rather these settings are safety mechanisms intended to protect against key stroke errors when delivering boluses and using temporary basal rates.

**Table 1:** Suggested AID setting changes when transitioning from U100 to U200 insulin in an AID system.

	Beta Bionics iLet		Medtronic 780G with Smart Guard		Insulet Omnipod 5		Tandem t:slim X2 and Mobi with Control-IQ	
	U100 Settings	U200 Settings	U100 Settings	U200 Settings	U100 Settings	U200 Settings	U100 Settings	U200 Settings
Factory reset needed when switching between insulin formulations?	Yes- algorithm is driven by TDI history		Yes- algorithm is driven by TDI history. Note: only one reset possible per pump lifetime		Yes- algorithm is driven by TDI history		No- Software Versions 7.9 and up does not reset TDI	
Weight (with examples)	Enter half of actual weight when switching to U200		n/a		n/a		No change needed- weight does not significantly impact insulin delivery	
	200lb	100lb						
Total Daily Insulin Dose (with examples)	n/a		n/a		n/a		Enter half of the total daily insulin dose when switching to U200	
							120 units	60 units
Basal rate (with examples)	n/a		Enter half the basal rate when switching to U200					
			e.g., 3.0 units/hr (U100), change to 1-1.5 units/hr (U200)					
Carbohydrate Ratio (with examples)	n/a		Double carbohyrate ratio when switching to U200					
			e.g., 1 unit per 5g carbohydrate (U100), change to 1 unit per 10-15g (U200)					
Correction/ Senstivity Factor (with examples)	n/a		Double the sensitivity when switching to U200					
			e.g., 1 unit per 10 mg/dL (U100), change to 1 unit per 20-30 mg/dL (U200)					
AID System-Specific Safety Settings *	n/a		Max Basal: when using U200 program half of the user’s U100 max basal (e.g. if U100 max basal was 4 units/hr, U200 max basal should be 2 units/hr) Max Bolus: when using U200 program 1/3 of the U200 TDI, 1/6 of the U100 TDI, or personalize according to typical U200 bolus amounts (e.g. if U100 TDI is 120 units and U200 TDI is 60 units, set max bolus to 20 units)					
Instructions for Factory Reset	Device Settings → “reset device” or “factory reset” (wording may vary depending on app version)		Settings menu → Device settings → Press and hold the right arrow key and the back key at the same time until the Manage Settings screen appears. Select ‘Clear Active Insulin’		Main menu → General settings. Note that factory reset erases all programmed insulin delivery settings.		n/a	
Considerations	iLet initiates insulin assuming a TDI of 0.6 units/kg/day		A factory reset can only be completed once, which could represent a significant limitation to U200 use with the 780G.		All insulin delivery profiles are erased with the factory reset.		Multiple profiles can be programmed, which allows users to program U100 and U200 profiles.	

Generally speaking, insulin delivery settings should be halved when transitioning from U100 to U200. Due to improved insulin absorption and/or decreased infusion site leaking, it may be advisable to decrease basal rates, carbohydrate ratios and correction factor/sensitivities by more than half in those with very high TDI.*For the AID systems described in this manuscript, the programmed maximum basal rate and bolus do not influence algorithm determined insulin delivery. Rather these settings are safety mechanisms intended to protect against key stroke errors when delivering boluses and using temporary basal rates. n/a, not applicable.

### Beta Bionics iLet

The iLet is unique in that standard pump settings are not programmed (no basal rates, carbohydrate ratios or correction/sensitivity factors); users do not enter carbohydrates for meal boluses, nor can they give a manual bolus to correct for hyperglycemia. At initiation, the iLet determines insulin dosing based on the user’s weight; over time, delivery is adapted according to insulin delivery history and glycemic patterns. For meals, the algorithm “learns” a bolus dose for each type of meal announced (Usual, More than Usual, Less than Usual). Therefore, it is vital to complete a factory reset to erase prior learning before switching from U100 to U200 in the iLet.

To complete a factory reset, under the settings menu, select “*Other*” followed by “*reset device*” or “*factory reset*” (wording may vary depending on app version). During the reset process, insulin delivery history and algorithm learning will be deleted from the device. The device will reboot automatically and then, the initial set up screens will appear, and users should enter half of their body weight (e.g. a user weighing 200lb, should enter 100lb) so the algorithm will assume half of their U100 TDI when the initiate the system with U200. The iLet assumes a TDI of 0.6 units/kg/day upon initiation; for PWD with a high TDI, consider their U100 TDI to calculate a body weight to enter that will better support the algorithm in meeting individual insulin delivery needs.

### Omnipod 5: automated mode

The Omnipod 5 AID algorithm uses TDI history to drive basal automation in automated mode. Adaptive basal rates are calculated as 50% of the TDI using a weighted average of TDI from the last 4–5 pods. The controller or app must be reset when changing between U100 and U200 to ensure the algorithm is not dosing according to previous TDI history.

Users can complete a factory reset in the main menu under ‘*General Settings*’. The reset must be completed when there is no active pod in use. This will erase algorithm learning from previous TDI history and delete all programmed settings.

### Medtronic 780G: SmartGuard

Similar to Omnipod 5, the 780G SmartGuard algorithm also uses TDI history to drive basal automation. Therefore, TDI history must be reset when switching from U100 to U200. It is important to note that the TDI history can only be cleared once. If ‘*Clear Active Insulin*’ does not display in the ‘*Mange Settings*’ menu, it is because the active insulin has previously been cleared and cannot be cleared again. This nuance to the 780G may represent a significant limitation in using U200 with this system. Additionally, because a factory reset deletes the TDI history and because 48 hours of insulin delivery history is required before activating SmartGuard, users will need to use the 780G in manual mode for at least 48 hours before being able to activate SmartGuard.

A factory reset can be completed in the settings menu. Select ‘*Device Settings’* in the settings menu. Then press and hold the right arrow key and the back key at the same time until the *Manage Settings* screen appears. In ‘*Manage Settings’* select ‘*Clear Active Insulin*’. This will clear the TDI and any current active insulin tracking from the pump.

### Tandem t:slim X2 and Mobi: Control-IQ

Although Control-IQ relies less on TDI history than other algorithms, TDI does impact automated insulin delivery to a lesser extent, and it is still advisable to update settings. TDI is programmed into the Control-IQ algorithm and is primarily used for minimum and maximum insulin delivery constraints in the algorithm. All software versions since the upgrade to Control-IQ+ (Version 7.9) do not reset TDI and so no factory reset is needed. When using U200, halve the individual’s TDI. For example, if the TDI on U100 insulin is 200 units, enter 100 units. The programmed weight does not significantly impact insulin delivery.

Two personal profiles should be programmed for U100 and U200 to facilitate transition between the two insulin concentrations. We suggest naming the profile U100 and U200 to ensure the correct settings are used with each insulin.

## Potential risks to U200-AID

While there are many potential benefits to U200-AID, it is important to consider the potential risks associated with this therapeutic approach that is not currently approved by any regulatory body. Off-label prescribing may create challenges with insurance and/or health system coverage, that could lead to gaps in access to therapy and/or additional costs for PWD along with additional administrative burden for HCPs. The lack of educational resources about U200-AID may complicate education for both PWD and HCPs, which could in turn lead to errors in programmed insulin delivery settings that may lead to a two-fold over- or under-dose of insulin and result in adverse events such as DKA or hypoglycemia. U200-AID use in adolescents may prove particularly challenging as this cohort takes on increasing responsibility for diabetes self-management and transitions from pediatric to adult care. Even if PWD and their diabetes care team are familiar with U200-AID, PWD often interact with non-diabetes HCPs as part of other out-patient medical care, inpatient care, emergency situations, and school and/or institutional settings. These risks should be explicitly discussed with U200-AID users, both to ensure they understand the off-label therapy they are accepting and to support them in recognizing the importance of contacting their diabetes care team when necessary.

## Discussion

U200-AID may help mitigate barriers to AID use by increasing the wear time of insulin pump cartridges, improving insulin absorption, and decreasing cost. We urge clinicians to be mindful of the impact of high TDI on AID use and its implications for both glycemia and quality of life for PWD. The cost of devices and diabetes supplies are among the most common barriers to diabetes technology use (20–22), and these barriers are exacerbated for PWD with high TDI requirements who require more insulin and pump supplies. The on-body presence of insulin pumps is commonly cited as another major barrier to AID use (20, 21). While engineering advances have the potential to allow for the development of smaller insulin pumps, these pumps would have smaller cartridge volumes and require more frequent device changes. U500 human insulin has been studied in open loop insulin pumps (23), however its pharmacokinetic and pharmacodynamic profile differ significantly from U100 (24, 25). Whereas U500 human insulin has an action time of 18.1-21.5 hours, the action time for U100 is 4–6 hours (24, 25) which limits its use in AID. Ongoing research has shown that faster-acting formulations of U500 rapid acting insulins (AT278 U500) are possible (26), and in the future these may be considered for use in AID. This paper provides practical considerations and guidance for clinicians on U200-AID use with an emphasis on patient education and methods to support safe use. While studies using real-world data provide preliminary evidence for safe use of U200 in AID, further prospective research and consideration by regulatory authorities are needed.

## Data Availability

The original contributions presented in the study are included in the article/supplementary material. Further inquiries can be directed to the corresponding author.
